# Impact of 4-week of a restricted Mediterranean diet on taste perception, anthropometric, and blood parameters in subjects with severe obesity

**DOI:** 10.3389/fnut.2023.1196157

**Published:** 2023-08-25

**Authors:** Camilla Cattaneo, Sara Paola Mambrini, Luisa Gilardini, Massimo Scacchi, Ella Pagliarini, Simona Bertoli

**Affiliations:** ^1^Sensory & Consumer Science Lab (SCS_Lab), Department of Food, Environmental and Nutritional Sciences (DeFENS), University of Milan, Milan, Italy; ^2^International Center for the Assessment of Nutritional Status (ICANS), Department of Food, Environmental and Nutritional Sciences (DeFENS), University of Milan, Milan, Italy; ^3^Istituto Auxologico Italiano, IRCCS, Laboratory of Metabolic Research, S. Giuseppe Hospital, Piancavallo, Italy; ^4^Istituto Auxologico Italiano, IRCCS, Obesity Unit – Laboratory of Nutrition and Obesity Research, Department of Endocrine and Metabolic Diseases, Milan, Italy; ^5^Department of Clinical Sciences and Community Health, University of Milan, Milan, Italy

**Keywords:** taste acuity, hypocaloric balanced Mediterranean diet, taste-oriented nutritional reeducation, salt recognition threshold, obesity, weight loss program

## Abstract

**Introduction:**

The study of taste functionality and its relation to human health is receiving growing attention. Obesity has been reported to cause alterations in sensory perception regarding system functionality and preferences. However, a small body of research addresses tastes perception and its modification with the achievement of body mass reduction through surgical intervention. Much fewer efforts have been made to evaluate the impact of mild restrictive nutritional intervention on gustatory functions. Thus, the objectives of this study were to determine if a dietary intervention of 4 weeks following a restricted balanced Mediterranean diet would affect the sweet and salty taste thresholds of subjects with severe obesity and could influence their anthropometric and blood parameters.

**Methods:**

Fifty-one patients with severe obesity (*F*: 31; age: 43.7 ± 12.5; BMI = 47.6 ± 1.0) were enrolled in the study. The recognition threshold for sweet and salty taste and anthropometric and blood parameters were assessed before and after the 4-week weight loss program.

**Results and Discussion:**

The Mediterranean diet has proven to be an effective treatment, significantly improving all anthropometric and blood parameters (*p* < 0.05) after 4 weeks of intervention. Moreover, the hypo-sodium treatment associated with the diet significantly improved the salty threshold (*p* < 0.001). No changes were detected for the sweet threshold. Collectively, these data highlight that dietary treatment might impact taste perception differently. Therefore, a taste-oriented nutritional intervention could represent a novel approach to developing more individualized, taste-oriented follow-up interventions to maintain sustainable and long-term weight loss.

## 1. Introduction

During the past three years, our world has been facing the coronavirus pandemic, and the loss of or alterations to taste and smell are one of the main symptoms of viral infection. Nowadays, more than ever before, clinicians and researchers in the field have recognized the importance of understanding, researching, and assessing sensory science for the promotion of health and the prevention of diseases (1). Thus, the need to improve chemo-sensation knowledge and perception in specific targets of consumers or patients fostered the application of psychophysiological studies to evaluate the senses of smell and taste quantitatively in various clinical and community settings through advances in sensory science.

Among various chronic diseases, obesity has been reported to cause alterations in sensory perception regarding system functionality and preferences (2). Indeed, individuals with obesity are reported to perceive tastes as less intense (3–6) and may need greater stimulation of taste and oral somatosensory systems to satisfy their reward, increasing the willingness to ingest energy-dense foods, particularly rich in sugars, fats, and salt. Indeed, several studies have emphasized the inverse relationship between the perception of sweet taste, salt taste and fat stimulus and nutritional status [e.g., (7–11)]. However, a small body of research addresses tastes perception and its modification with the achievement of body mass reduction through dietary or surgical intervention. While literature findings underscored a more robust and sustained impact of bariatric surgery in affecting taste perception (12, 13), much fewer efforts have been made to evaluate the impact of mild restrictive nutritional intervention on gustatory functions. It is still unclear if the changes in taste perception are a surgery-specific phenomenon or if it is a general phenomenon that accompanies weight loss. Indeed, the data concerning this topic still need to be more consistent (14). A link between diet and fat taste has been shown in two intervention studies, whereby the modification of fat content of the diet (i.e., low-fat dietary intervention) induced a significant weight reduction and positively influenced fat stimulus threshold, resulting in a decreased taste threshold (increased sensitivity) in both lean participants and those with obesity (15) and overweight and obese subjects (16). However, in this previous study (16), the low-fat diet consumption over the 6 weeks had no significant positive effect on sweet and salty detection thresholds.

On the contrary, the sweet taste threshold was decreased in women with obesity after diet-induced weight loss programs of 3 months (17, 18). Because data are limited and conflicting, it is therefore important to understand whether taste perception may change during a weight-loss program and how this may contribute to the success or failure of achieving a long-term dietary modification. Hypothetically, an individual following a taste-oriented diet could experience an improvement in taste system functionality, which may help to modify taste preferences and accomplish healthier dietary habits.

In 2010, the Mediterranean diet was awarded the recognition of UNESCO as an Intangible Heritage of Humanity (19). Several studies and guidelines indicate the Mediterranean diet as the non-pharmacological dietary approach of choice in the management of patients with severe obesity (20, 21). Literature data show that the Mediterranean diet has a protective role against non-communicable diseases and several benefits by shutting down low-grade inflammation. It is characterized by high consumption of whole cereals, fruit, legumes, vegetables, and nuts, a moderate use of dairy products, a low consumption of meat and poultry and a moderate consumption of alcohol. Its high content of plant and whole foods has been shown to increase satiety, and this may also improve adherence in individuals who need to lose weight on a hypocaloric diet (21, 22). As also shown in some studies, diets unbalanced in favor of some nutrients rather than others (e.g., low fat, high protein, or low carbohydrates) do not seem to be more effective than a balanced, moderately low-calorie diet as Mediterranean pattern (23, 24). This dietary pattern is characterized by a low content of processed foods, favoring the consumption of less processed foods with a lower content of sodium, simple sugars, and saturated fats instead. Moreover, several epidemiological and clinical studies have highlighted the positive effects of the Mediterranean diet on cardiovascular risk factors, obesity, metabolic syndrome, non-alcoholic fatty liver disease, and diabetes (21, 25–28).

Based on these premises, scientists and clinicians can utilize this information to develop more individualized, taste-oriented follow-up interventions to maintain sustainable and long-term weight loss, with the perspective of more personalized and precise nutritional therapy. Thus, the primary goal of this study was to determine if a weight loss dietary intervention of 4 weeks following a restricted balanced Mediterranean diet would affect the sweet and salty taste thresholds of subjects with severe obesity. Moreover, the second aim of the present study was to ascertain whether and to what extent dietary intervention could influence anthropometric and blood parameters.

## 2. Materials and methods

### 2.1. Patients recruitment

This study was carried out as part of a larger study (11), which aims to identify predictors that may play a role in successful weight loss and maintenance in obese subjects with eating disorders.

The study protocol was performed according to the principles established by the Declaration of Helsinki and approved by the Istituto Auxologico Italiano ethics committee (Approval registration number: 43C101). Written informed consent was obtained from all participants before entering the study.

The general study population consisted of adults of both sexes with severe obesity who self-referred to Istituto Auxologico Italiano and later enrolled as part of a 4-weeks weight loss program. Participants had to meet the following criteria: body mass index (BMI) > 30 kg/m^2^ and 18–60 years of age. Participants were excluded if they were uncooperative, pregnant or breastfeeding, heavy smokers, had undergone bariatric surgery, or had a medical condition that affected their taste or weight-loss ability (i.e., thyroid disorders, presence of endocrine abnormalities associated with obesity, current or recent oral, nasal or sinus infections, major psychiatric disorders) (11). At the end of the recruitment phase, a total of fifty-one subjects (F: 60.7%) with severe obesity (BMI = 47.6 ± 7.2 kg/m^2^) and a mean age of 43.7 ± 12.5 years were enrolled in the study.

### 2.2. Study outline

This study was a dietary intervention where participants, admitted for residential rehabilitation hospitalization, followed a hypocaloric balanced Mediterranean diet. All participants were required to attend one laboratory session at baseline and week 4, during which general condition, blood, and anthropometric measures were examined, and detection threshold tests for sucrose and sodium chloride using ascending forced choice triangle tests (29) were evaluated. Moreover, participants were required to complete the Binge Eating Scale (BES) (30) to assess the presence and severity of binge eating disorders.

**Figure 1:**
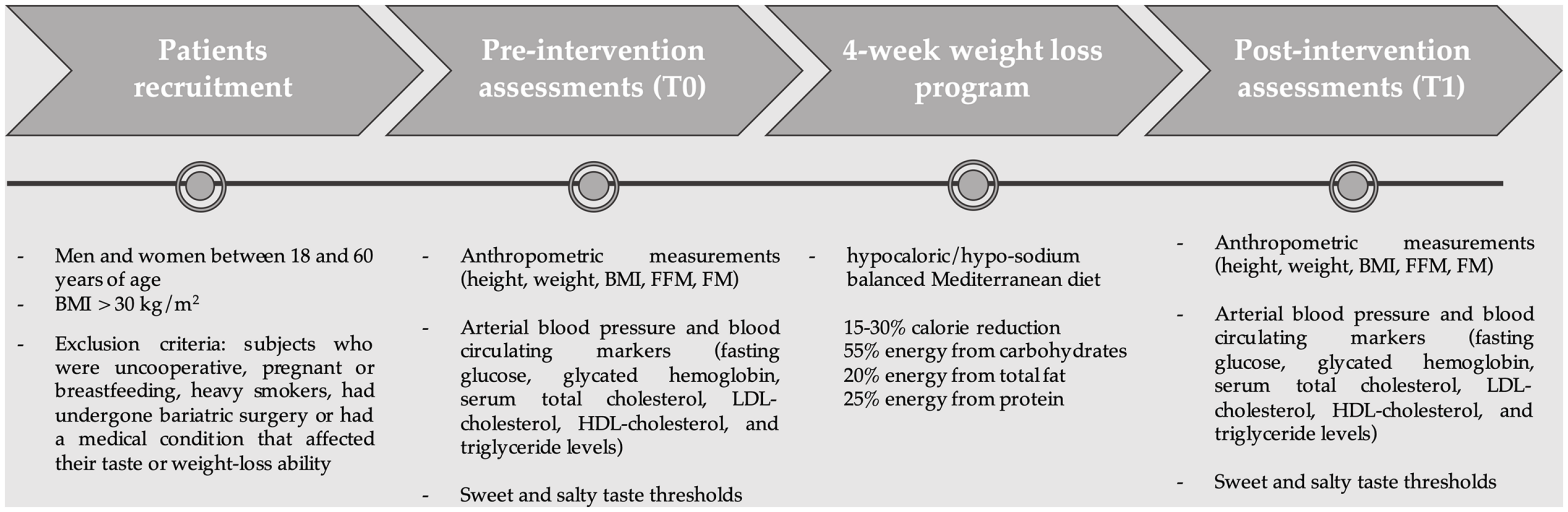
Study flow diagram.

Figure 1 illustrates the flow of the study.

#### 2.2.1. Weight loss program

The 4 weeks weight loss program took place at Istituto Auxologico Italiano where the patients were admitted for rehabilitation hospitalization. Before receiving the dietary prescription, the patients underwent a complete nutritional and dietary assessment to evaluate their dietary habits. The trained dieticians generally reported a dietary pattern rich in ultra-processed foods higher in salt, sugars, and fats. Following the dietary assessment, a dietary plan is set that considers nutritional needs in relation to the subjects’ basal metabolism calculated with Mifflin (31).

The patients were asked to follow the 4-week weight loss program (i.e., a personalized, restricted, balanced Mediterranean diet) (32). The hospital provided the meals, and generally, the dietary plan consisted of a calorie reduction of 15–30% of the usual intake, which was considered effective in ensuring adequate weight loss over time, with a macronutrient distribution based on the Mediterranean pattern (55% energy from carbohydrates, 25% energy from total fat, and 20% energy from protein). Table 1 shows the bromatological composition of the dietary intervention (3 dietary plans are reported as examples to show that the diet was personalized for each patient). The average dietary sodium content was 2000 mg sodium, corresponding to less than 5 grams of sodium chloride. In contrast, the diet’s average content of simple sugars is about 10–12% of the total kcal. An example of a weekly dietary plan is reported in Supplementary Table S1.

**Table 1:** Example of bromatology composition of the restricted Mediterranean diet for three subjects.

	Energy (kcal)	Protein (g)	Protein (%)	Lipid (g)	Lipid (%)	Glucides (g)	Glucides (%)
Subject 1	1,500	71	19	43	26	210	55
Subject 2	1,650	75	18	45	24	240	58
Subject 3	1800	94	20	51	25	255	55
Average of all dietary plans	1,655 ± 205 ^a^	79 ± 13	19	46 ± 4	26	228 ± 34	55

The average of all dietary plans of all patients involved has been reported as a mean ± standard deviation.

A dietary approach of this type, combined with nutritional counseling, is associated with objectives such as adequate weight loss, consumption of complete and proper meals, acquiring and reinforcing correct eating habits, and improving recognition of the biological stimuli of hunger and satiety in the timing of meal consumption (33).

#### 2.2.2. Anthropometric measures, biochemical analysis, and clinical evaluations

Using standard techniques, anthropometric measurements were performed after an overnight fast. At baseline, height (m) was measured to the nearest 0.1 cm using a SECA 217 vertical stadiometer (SECA, Hamburg, Germany). Body weight was measured to the nearest 100 g using a SECA 700 scale at baseline and week 4. BMI was calculated as weight (kg)/height (m^2^) and classified according to the WHO cut-offs (34). Body composition [fat mass (FM) and fat free mass (FFM)] was assessed by Bioimpedance (BIA). The blood sample, analyzed during routine laboratory, was collected in a fasting state with a venous sampling and stored at −80°C until the analysis took place with the oxidase enzymatic method measured using a Cobas Integra 800 Autoanalyzer (Roche Diagnostics, Monza, Italy) for glucose. Colorimetric enzymatic assays measured using a Cobas Integra 800 Autoanalyzer were used to determine serum total cholesterol (Roche Diagnostics, Monza, Italy reference code 03039773190), LDL-cholesterol (Roche Diagnostics, Monza, Italy reference code 07005717), HDL-cholesterol (Roche Diagnostics, Monza, Italy reference code 07528566), and triglyceride levels (Roche Diagnostics, Monza, Italy reference code 20767107). Blood pressure was measured at baseline and week 4 using a manual sphygmomanometer. The clinical pharmacological examinations were performed on the same day. Based on blood and anthropometric parameters, for each subject, obesity-related comorbidities were defined as such: Hypertension [SBP ≥140 mm Hg; DBP ≥90 mm Hg; or use of antihypertensive drugs (35)]; Diabetes (fasting plasma glucose ≥126 mg/dL; or use of oral anti-glycemic medication or insulin (36); Dyslipidemia [HDL-cholesterol <35 mg/dL; triglycerides >200 mg/dL, (36)]; Metabolic syndrome (HDL-cholesterol <40 mg/dL in men and < 50 mg/dL in women, triglycerides ≥150 mg/dL, SBP ≥130 mm Hg or DBP ≥85 mm Hg, fasting plasma glucose ≥100 mg/dL. MS was defined as 3 or more of the above components (37). Participants with a BES score ≥ 18 were identified as binge eaters (30).

#### 2.2.3. Salt and sweet thresholds

The detailed protocol was fully described in (11). In brief, the test was carried out according to International Organization for Standardization (29) using triangle tests with ascending forced choice methodology. Sweet and salty taste acuity was evaluated using filter paper strips (Indigo Instruments – Cat#33814-Ctl; 47 × 6 × 0.3 mm) immersed in ten aqueous solutions with increasing concentrations of sucrose and sodium chloride, respectively. The best-estimated threshold was used for each participant to determine salty and sweet recognition thresholds (29).

### 2.3. Statistical analysis

#### 2.3.1. Sample size calculation

A power calculation was conducted to determine the appropriate sample size for the study. Using data from a pilot study and previous research (16, 18), to detect a threshold difference of 0.2 mM sodium chloride between baseline and week 4, a sample size of *n* = 41 obese subjects will allow testing for medium effect sizes of *d* = 0.4 (repeated-measures t-test, α = 0.05, β = 0.8) as calculated with G-power (version 3.13).

#### 2.3.2. Data analysis

Data in the tables and figures are presented as mean ± SD unless otherwise indicated or medians with interquartile range (IQR = 75th − 25th percentile) for skewed data sets. Sucrose and NaCl detection thresholds were positively skewed and required logarithmic transformation to approximate a normal distribution. Bivariate correlations such as Pearson/Spearman correlation coefficients were used to determine whether there was a relation between taste thresholds and blood and anthropometric parameters. The bivariate correlations were performed at baseline (t0), and to know if the correlation changes after 4 weeks, a delta Δ (value at T1 – value at T0) was calculated for each variable and then correlated. McNemar’s test was used to compare the number of subjects presenting comorbidities related to obesity pre- and post-intervention. Paired *t*-tests were used to analyze changes in sucrose and NaCl oral detection thresholds from baseline to week 4. The Wilcoxon signed-rank tests were used to analyze changes in blood and anthropometric measurements between baseline and week 4. All analyses were performed with SPSS software (version 27.1, IBM, Armonk, New York), and the criterion for statistical significance was *p* ≤ 0.05.

## 3. Results

### 3.1. Characteristics of subjects

The general characteristics of the subjects are reported in Table 2. A total of 51 subjects with severe obesity (BMI = 47.6 ± 7.2 kg/m^2^) and a mean age of 43.7 ± 12.5 years were enrolled in the study. 60.7% of the subjects were female, and 27.4% were smokers.

**Table 2:** General characteristics of the evaluated subjects (*n* = 51).

Variables	Values
*Gender (n; %)*	
*F*	31; 60.7%
M	20; 39.3%
*Age* (*mean* ± SD)	43.7 ± 12.5
*Smoking status (n; %)*	
Yes	14; 27.4%
No	37; 72.6%
*Comorbidities*	
Hypertension, (*n*; %)	41; 80.4%
F:M (*n*)	24:17
Diabetes, (*n*; %)	12; 23.5%
F:M (*n*)	6:6
Dyslipidemia, (*n*; %)	6; 11.8%
F:M (*n*)	1:5
Metabolic syndrome, (*n*; %)	21; 41.2%
F:M (*n*)	13:8
Binge eating disorder, (*n*; %)	15; 29.4%
F:M (*n*)	11:4

### 3.2. Significant correlation between blood and anthropometric parameters and salt and sweet taste thresholds

**Figure 2:**
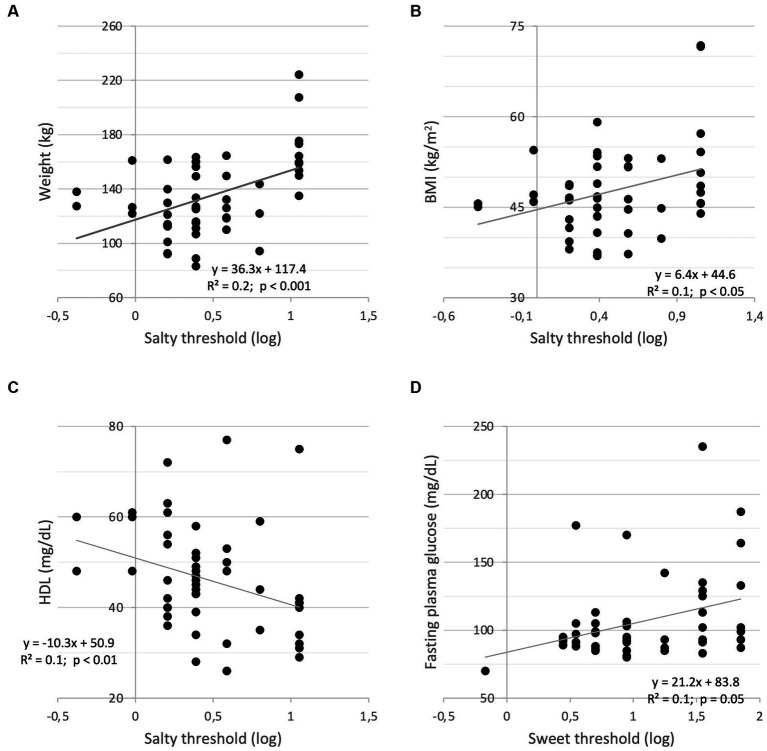
Correlations between salty threshold (log) and weight **(A)**; BMI **(B)**; HDL-cholesterol **(C)**; Correlation between sweet threshold (log) and fasting plasma glucose **(D)**.

As far as the salty threshold is concerned, statistically significant correlations with anthropometric parameters are observed at the baseline. Indeed, weight (*r* = 0.47, *p* < 0.001) and BMI (*r* = 0.33, *p* < 0.05), correlated positively and significantly with the recognition threshold (Figures 2A,B, respectively), indicating that higher weight and BMI correspond to an increased perception threshold of the salty taste (i.e., a reduced sensitivity). A negative and significant correlation between the salt threshold and the levels of circulating high-density plasma lipoprotein (HDL-cholesterol, ρ = − 0.40, *p* < 0.01; Figure 2C).

No significant correlations were observed for the sweet threshold besides the positive and significant correlation with fasting plasma glucose (ρ = 0.27, *p* = 0.05; Figure 2D), indicating that a reduced sweet sensitivity corresponds to higher concentrations of plasma glucose in the fasting state.

To investigate whether the correlation changes after 4 weeks, bivariate correlation analyses were performed showing that changes in salty threshold positively correlates with changes in Δweight (*r* = 0.29, *p* < 0.05), ΔBMI (*r* = 0.34, *p* < 0.05), Δ%FM (ρ = 0.33, *p* < 0.05) and negatively with Δ%FFM (ρ = 0.33, *p* < 0.05). In addition, changes in sweet threshold positively correlated with fasting plasma glucose (ρ = 0.25), albeit not significantly (*p* = 0.08).

### 3.3. Significant amelioration of blood and anthropometric measures after the dietary intervention

During the dietary intervention, body weight (*p* < 0.001) and BMI (*p* < 0.001) decreased significantly in a manner similar to fat mass (*p* < 0.001). On the contrary, fat-free mass significantly increased (*p* < 0.001). Both systolic and diastolic blood pressure also improved significantly (*p* ≤ 0.001). Moreover, the program was sufficient to determine a statistically significant improvement in all the blood parameters considered.

As regards the comorbidities related to obesity, the dietary intervention significantly reduced the number of subjects who presented values above the cut-off after the restricted Mediterranean diet (post-intervention: Hypertension *n* = 9, *χ*^2^ = 26.4, *p* < 0.001; Diabetes *n* = 4 *χ*^2^ = 12.7, *p* < 0.01; Dyslipidemia *n* = 1, *χ*^2^ = 8.6, *p* = 0.12; Metabolic syndrome *n* = 5, *χ*^2^ = 9.0, *p* < 0.001).

The anthropometric and blood characteristics of all the subjects pre- (baseline) and post- (4-weeks later) dietary intervention are reported in Table 3.

**Table 3:** Subjects’ anthropometric and blood characteristics pre– (baseline) and post– (4-weeks later) dietary intervention.

Parameters	Pre-intervention	Post-intervention	*p*-values
*Anthropometric data*			
Weight (kg)	134.7 (4.0)^a^	127.4 (3.7)	< 0.001
Body mass index (kg/m^2^)	47.6 (1.0)	45.1 (0.9)	< 0.001
Fat mass (%)	53.8 (0.07)	52.5 (0.07)	< 0.001
Fat free mass (%)	46.1 (0.06)	47.5 (0.06)	< 0.001
*Arterial blood pressure*			
Systolic blood pressure (mmHg)	137.5 (88.0–106.0)^a^	130.0 (120.0–130.0)	< 0.001
Diastolic blood pressure (mmHg)	80.0 (80.0–90.0)	80.0 (70.0–80.0)	0.001
*Blood chemistry*			
Fasting plasma glucose (mg/dL)	94.0 (88.0–106.0)	94.0 (84.0–101.0)	< 0.001
Glycate hemoglobin (mmol/mol)	39.0 (34.75–45.25)	37.0 (33.0–42.0)	< 0.001
Total cholesterol, mg/dL	183.6 (149.0–205.2)	154.2 (128.2–172.4)	< 0.001
HDL-cholesterol, mg/dL	44.0 (39.0–53.0)	41.0 (34.0–47.0)	< 0.001
LDL-cholesterol, mg/dL	109.0 (88.0–131.0)	82.0 (70.0–101.0)	< 0.001
Triglycerides, mg/dL	127.0 (95.0–191.0)	118.0 (86.0–165.0)	0.04

a Data are presented as mean ± SEM or medians with IQR.

### 3.4. Impact of the dietary intervention on taste thresholds

**Figure 3:**
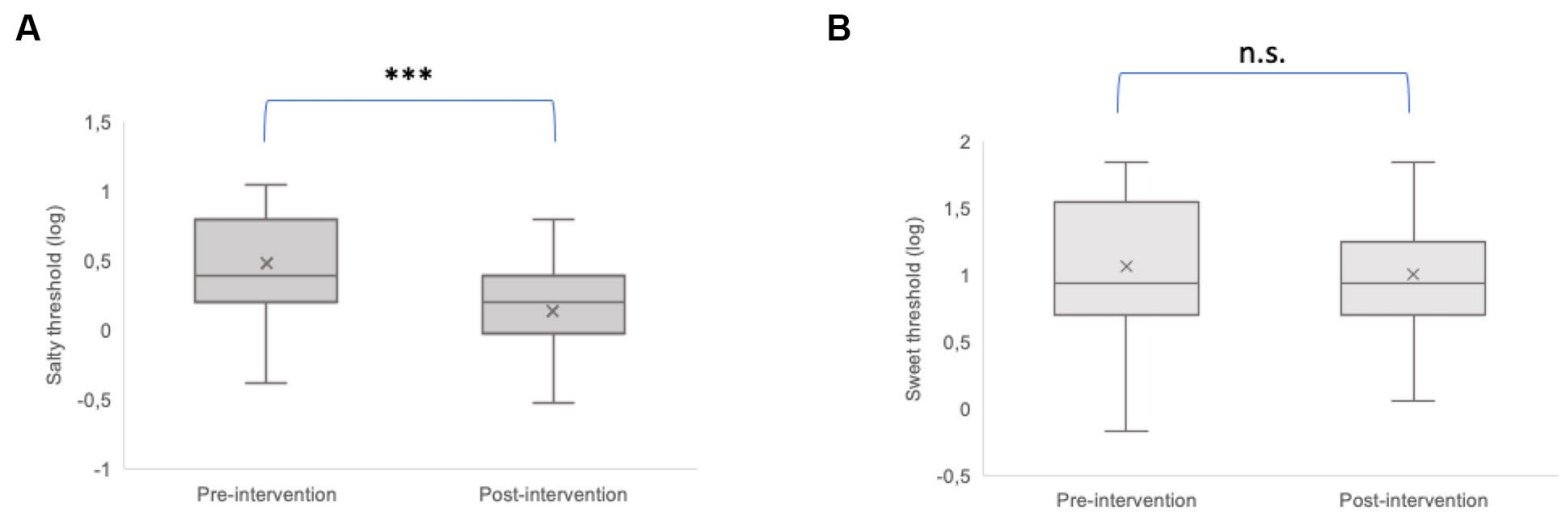
Comparison of the taste threshold of saltiness **(A)** and sweetness **(B)** before (pre-intervention) and after (post-intervention) weight loss intervention. The plots provide a representation of data distribution (the ‘box’), the minimum and the maximum (the ‘whiskers), and the median (horizontal line) ± IQR (vertical line) within each group. Statistics according to *t*-test: ***p < 0.001; n.s. not significant.

The taste thresholds of saltiness and sweetness before and after dietary intervention are reported in Figure 3. There was a significant decrease in salt taste thresholds following the consumption of the hypocaloric balanced Mediterranean diet (*p* < 0.001) (Figure 3A). In contrast, the weight loss program had no significant effect on sweet thresholds (*p* = 0.39) (Figure 3B).

## 4. Discussion

This study aimed to assess whether a weight loss dietary intervention (i.e., restricted balanced Mediterranean diet) in people with severe obesity can modify various factors (i.e., anthropometric, blood, and sensory parameters), which are generally negatively affected by the pathological condition. It is well known that anthropometric parameters and lipid profile changes were more evident in subjects with severe obesity. Moreover, it has been suggested that obesity seems to affect taste functionality (2), probably due to an inflammatory response in the fungiform taste buds, which has been correlated with impairments in taste perception (38).

In the present study, this relationship was studied by analyzing the influence of anthropometric and blood parameters of patients with severe obesity on the thresholds of sweet and salt taste stimuli. The results at baseline showed a general increase in salty taste threshold (i.e., decreased acuity to saltiness) corresponding to an increase in weight and BMI, supporting the hypothesis that obese adults consume more salty foods and have reduced salt sensitivity and higher salt preference (39, 40). On the contrary, a negative correlation was found between HDL levels and salty taste recognition thresholds, in accordance with previous results (37, 41). Moreover, we found a direct correlation between fasting plasma glucose levels and sweet taste recognition threshold, suggesting a blunted sweet taste response in this group of subjects with severe obesity. Accordingly, an increase in taste thresholds has been previously associated with hyperglycemia and insulin resistance (42, 43). Noteworthy, these correlations also remain after 4 weeks when changes (Δ) were analyzed. In particular, the decrease in salty threshold is significantly correlated with a decrease in anthropometric parameters (i.e., weight, BMI, fat mass and fat-free mass), such that the greater the decrease in 4-weeks intervention, the greater the decrease in parameters related to obesity.

As regards the weight loss dietary intervention in patients with severe obesity, the restricted Mediterranean diet has proven to be an effective treatment, showing an improvement in all anthropometric and blood parameters after 4 weeks of intervention. Our results confirmed the efficacy of the dietetic treatment in ameliorating many risk markers associated with obesity, cardiovascular diseases, and metabolic syndrome (28, 44–46). The adequate consumption of plant and whole foods and unsaturated fatty acids and the synergic effect and mechanisms of specific nutrients have had a direct impact on all risk markers evaluated, namely, BMI, blood pressure, fasting blood glucose, high-density lipoprotein (HDL) cholesterol, and triglycerides, as well as systemic inflammation that characterized subjects with severe obesity. The adherence to the Mediterranean diet could have facilitated these interesting results. Indeed, recent research has shown that greater adherence to the Mediterranean diet is effective for prevention and management of different diseases and is associated with a significant improvement in health status (25, 47–49). In the present study, the residential and controlled inpatient setting have guaranteed a greater adherence of the patients to the dietary treatment. Nevertheless, the hypo-sodium treatment associated with the diet (average sodium content between 1,500 and 2000 mg per day) significantly improved salt thresholds. Indeed, the interesting finding of this study is that salty taste thresholds significantly decreased (i.e., increased sensitivity to sodium chloride) after the 4-week diet. Our results align with previous findings (50), whereby one week of sodium restriction improves the taste threshold for the salty taste in patients with chronic kidney disease and healthy subjects. On the contrary, the study of Newman and colleagues (16) did not report such improvement. This discrepancy could be due to the type of dietary treatment chosen (i.e., fat-taste-oriented) since the low-fat intake provided in (16) specifically affected and improved the fat-taste thresholds.

Although previous studies highlighted a positive effect of the dietary intervention on the thresholds (18, 51) and preferences (52) for sweet taste, no modifications in sweet taste thresholds - corresponding to changes in consumption of sugars and carbohydrates from baseline to the end of dietary intervention - were highlighted in the present study. A possible explanation for the conflicting results may lie in the different duration of dietary treatments since all the cited studies planned dietary interventions longer than 3 months (i.e., from 12 to 30 weeks). Seeing as how differently the hypocaloric balanced Mediterranean diet affected the sweet and salty thresholds, we can speculate that this discrepancy could be due to the different trans-duction mechanisms related to the taste receptors for salt and sweet (sodium channel vs. G-protein coupled receptors, respectively). The decreases in salty taste threshold are specific to the sodium restriction throughout the four weeks. They could be due to changes in the background concentration of salivary Na + to which the taste receptors are adapted, which is much easier to achieve than a modification in sweet-sensitive type II receptor cells (53). Thus, it is possible that the dietary approach proposed in the present study would need to be followed over a more extended period of time before definitive changes in sweet taste thresholds would be seen.

Nevertheless, our preliminary results seemed promising and could greatly impact an individual’s dietary behaviors. Indeed, it is noted that a reduction in the usual amount of oral sodium intake is one of the candidate factors influencing the recognition threshold for salty taste (54). Also, the preference for salty foods may change with an acute transient increase in salt preference. Then, after weeks or months on a low-sodium diet, a shift in the opposite direction was reported (55).

To the best of our knowledge, this is the first study that assessed the effects of the restricted balanced Mediterranean diet on salt and sweet taste thresholds in patients with severe obesity. Investigating whether taste functionality in this kind of patient might be improved by dietary intervention would increase understanding of how to develop more precise taste-oriented nutritional therapy and improve the compliance of these subjects to the treatment.

A limitation of this work lies in the duration of the treatment. This study examined the effects of short-term weight loss on eating behavior and taste perception. However, future work should consider investigating the efficacy of dietary treatments of longer duration, which may lead to better results on both salty and sweet taste thresholds. In addition, even if the sample size was appropriate to observe a moderate effect size, the number of participants is still limited, and no separation by sex or age could be performed. Moreover, for simplicity and design clarity, only two taste stimuli (i.e., NaCl and sucrose) were used in the current study and presented using filter paper strips. Still, future studies should evaluate other taste stimuli (e.g., sour, bitter, and fat) and examine the effects of dietary intervention on taste perception and preferences using more ecologically relevant food stimuli (i.e., solid stimuli containing texture and smell in addition to taste). Finally, our study cannot determine whether taste perception and eating behavior in our obese subjects were different from lean subjects because a control group in the study design was not included.

## 5. Conclusion

In conclusion, our results showed an improvement in taste thresholds of saltiness, thanks to a re-education of the taste buds through a controlled sodium diet. This approach led to a better taste perception, improving its sensitivity by approaching normal values. It can be hypothesized that the weight loss will continue following the dietary plan after hospitalization, and taste thresholds may also improve further. This would lead to good dietary compliance over time, as the hedonistic satisfaction first sought through highly palatable foods can also be satisfied with consuming healthier foods with lower caloric density. Therefore, targeting the taste could represent a new approach to weight control to prevent the risk of therapeutic failure and identify new personalized intervention strategies.

## Data availability statement

The raw data supporting the conclusions of this article will be made available by the authors, without undue reservation.

## Ethics statement

The studies involving humans were approved by Istituto Auxologico Italiano Ethics Committee (Approval registration number: 43C101). The studies were conducted in accordance with the local legislation and institutional requirements. The participants provided their written informed consent to participate in this study.

## Author contributions

CC, SM, EP, and SB: conceptualization, methodology, and resources. CC and EP: formal analysis. CC and SM: investigation and writing—original draft preparation. CC, SM, LG, MS, EP, and SB: writing—review and editing. CC: visualization. EP and SB: supervision and project administration. SB: funding acquisition. All authors contributed to the article and approved the submittedversion.

## Funding

This study was supported by Finanziamento Ricerca Corrente, Ministero della Salute.

## Conflict of interest

The authors declare that the research was conducted in the absence of any commercial or financial relationships that could be construed as a potential conflict of interest.

## Publisher’s note

All claims expressed in this article are solely those of the authors and do not necessarily represent those of their affiliated organizations, or those of the publisher, the editors and the reviewers. Any product that may be evaluated in this article, or claim that may be made by its manufacturer, is not guaranteed or endorsed by the publisher.
