# Supplementary material for: Impact of 4-week of a restricted Mediterranean diet on taste perception, anthropometric, and blood parameters in subjects with severe obesity
Source: Front Nutr. 2023 Aug 25;10:1196157. doi: 10.3389/fnut.2023.1196157 (PMC10485378; doi:10.3389/fnut.2023.1196157)
Supplement: Supplementary file 1 [file Data_Sheet_1.PDF]

Supplementary Table S1. Example of weekly dietary plan.

Weekday	Breakfast	Lunch	Dinner
**Monday**	Melba toast + milk or light yoghurt<br>Coffee or tea	Risotto with pumpkin<br>Omelette (with bread)<br>Cauliflowers<br><br>Seasonal fruit	Vegetable soup with pasta<br>Peas (with bread)<br>Zucchini<br><br>Seasonal fruit
**Tuesday**	Melba toast + milk or light yoghurt<br>Coffee or tea	Tomato pasta<br>Swordfish (with bread)<br>Salad<br><br>Seasonal fruit	Whole wheat pasta with vegetables<br>Roasted chicken (with bread)<br>Brussel sprouts<br><br>Seasonal fruit
**Wednesday**	Melba toast + milk or light yoghurt<br>Coffee or tea	Risotto with saffron<br>Roasted turkey (with bread)<br>Tomatoes<br><br>Seasonal fruit	Vegetable soup with barley<br>Beans (with bread)<br>Boiled fennel<br><br>Seasonal fruit
**Thursday**	Melba toast + milk or light yoghurt<br>Coffee or tea	Barley with seasonal vegetables<br>Cream cheese (with bread)<br>Artichokes<br><br>Seasonal fruit	Vegetable soup with rice<br>Boiled Peas (with bread)<br>Green beans<br><br>Seasonal fruit
**Friday**	Melba toast + milk or light yoghurt<br>Coffee or tea	Asparagus soup<br>Boiled chicken thigh (with bread)<br>Zucchini<br><br>Seasonal fruit	Whole wheat pasta with vegetables<br>Hake in lemon cream (with bread)<br>Broccoli<br><br>Seasonal fruit
**Saturday**	Melba toast + milk or light yoghurt<br>Coffee or tea	Vegetable soup with pasta<br>Boiled lentils (with bread)<br>Brussel sprouts<br><br>Seasonal fruit	Whole wheat pasta with tomato puree<br>Trout (with bread)<br>Carrots<br><br>Seasonal fruit
**Sunday**	Melba toast + milk or light yoghurt<br>Coffee or tea	Risotto with radicchio<br>Veal stew with tomato puree (with bread)<br>Tomatoes<br><br>Seasonal fruit	Whole wheat pasta with tomato puree and capers<br>Ricotta cheese (with bread)<br>Eggplant<br><br>Seasonal fruit
